# A Baseline Study of Oxygen Saturation in Parafoveal Vessels Using Visible Light Optical Coherence Tomography

**DOI:** 10.3389/fmed.2022.886576

**Published:** 2022-05-12

**Authors:** Jingyu Wang, Weiye Song, Natalie Sadlak, Marissa G. Fiorello, Manishi Desai, Ji Yi

**Affiliations:** ^1^Department of Ophthalmology, School of Medicine, Johns Hopkins University, Baltimore, MD, United States; ^2^School of Mechanical Engineering, Shandong University, Jinan, China; ^3^Department of Ophthalmology, Boston Medical Center, Boston, MA, United States; ^4^Department of Biomedical Engineering, Johns Hopkins University, Baltimore, MD, United States

**Keywords:** visible light optical coherence tomography, retinal oximetry, baseline study, parafoveal vessels, segmentation

## Abstract

The retinal macula is at the center of our visual field, and thus pathological damage in the macula significantly impacts an individual's quality of life. The parafoveal vessels form the inner retina provide oxygen perfusion, and the measurement of parafoveal oxygen saturation (sO_2_) can evaluate macular metabolism and provide pathophysiological insight. In this paper, for the first time, we present a baseline study of microvascular oxygen saturation (sO_2_) in perifoveal macular region using visible light optical coherence tomography (VIS-OCT) on normal eyes. The arterial and venous sO_2_ from all eyes was 92.1 ± 7.1 (vol %) and 48.4 ± 5.0 (vol %) (mean ± SD), respectively. Arteriovenous sO_2_ difference was 43.8 ± 9.5 (vol %). Marginal correlation was found between venous sO_2_ and intraocular pressure (IOP) among eyes. No significant correlation was found between sO_2_ and vessel topological features, including length, diameter, and distance to fovea. This baseline study could serve as a benchmark for the future sO_2_ investigation of retinal macular pathologies.

## Introduction

Oxygen supply supports the metabolism of the human retina, and abnormal oxygen perfusion is associated with various retinal conditions leading to vision damage and blindness, including glaucoma, age-related macular degeneration, diabetic retinopathy, and vascular occlusions (1). Therefore, the measurement of blood oxygen saturation within the retinal circulation is not only essential to understanding the physiopathology of retinal diseases, but also can play important role for detection and monitoring.

Label-free optical retinal oximetry harnesses the oxygen-dependent spectral contrast of endogenous hemoglobin to non-invasively measure oxygen saturation quantitatively in the human retina (2–6). A fundus camera based multi-wavelength oximetry has been previously reported in several clinical studies on retinal conditions (7). However, the technique lacks depth resolution, and the measurement is complicated by the reflections from vessel surface. So far, only readings from large vessels in the peri-papillary region have been reported.

Visible light optical coherence tomography (VIS-OCT) is a recent development that provides the necessary 3D imaging capacity to eliminate confounding signals from other retinal layers and the choroid (8–12). The much stronger absorption in visible light range than conventional near infrared light (8), in conjunction with 3D segmentation of retinal blood vessels, permits reliable spatio-spectral analysis for microvascular retinal oximetry (11). By combining OCT angiography, capillary oximetry in the human retina has been recently reported which is a significant technical contribution. The initial report of *in vivo* oximetry using VIS-OCT was introduced in 2011 (13), then VIS-OCT oximetry was applied in rat's retina in 2013 (8). After several years of development, the technique has been successfully demonstrated in human retina in pathological cases for clinical research, including glaucoma, retinal ischemia, diabetic retinopathy (DR), central retinal vein occlusion (CRVO) and sickle cell retinopathy (SCR) (14–16).

Being responsible for the central vision field and acuity, macular region contains >30% of the total ganglion cells in the whole retina (17) in addition to being enriched with cone photoreceptors. The quantification of macular vascular sO_2_ has important implications for macular retinal function and metabolism. However, existing reports so far have evaluated the sO_2_ of large retinal vessel around the optic nerve head (ONH) (4, 18–20). Due to lack of resolution in fundus-based 2D oximetry, macular region vascular sO_2_ has not been sufficiently studied before. In this study, for the first time, we report a baseline study of sO_2_ of parafoveal arterioles and venules. We analyze the association of the arterial and venous sO_2_ with ophthalmic exam, and OCT macular and ONH scans. The result will serve as an important reference for the future comparison with pathological data.

## Materials and Methods

### Human Subjects

This study was conducted at Boston Medical Center, whose Institutional Review Board reviewed and approved the study. The study was compliant with the Health Insurance Portability and Accountability Act and adhered to the tenets of the Declaration of Helsinki. Written informed consent was obtained from all subjects before participation. Healthy subjects were recruited through the Boston Medical Center Optometry clinics. Cataracts were evaluated using the Lens Opacification System II based upon color and opalescence (21). The system uses a 4-point grading system with increasing number consistent with increasing maturity. Severe cataracts graded more than 2+ were excluded. All the subjects went through a regular ophthalmic examination including refractive error, tonometry for intraocular pressure (IOP), stereoscopic optic disc assessment, as well as clinical OCT scans of optic nerve head (ONH) and macula by Zeiss Cirrus OCT device. The OCT thickness of circumpapillary retinal nerve fiber layer (cpRNFL) and macular ganglion cell complex (GCC: ganglion cell layer + inner plexiform layer) were recorded, as well as cup to disc ratio (CDR). Dual-channel VIS-OCT was performed subsequently, and parafoveal vessel oxygen saturation (sO_2_) were quantified by post-processing. The subjects were imaged by trained technicians.

### Study Device and Method

The detailed setup of self-built vis-OCT system can be referenced in our previous publication (22). The pattern of raster scanning was 512 × 256 pixels and signals were received by a line camera with a line rate of 50 kHz and an exposure time of 19.1 μs. The image field of view (FOV) was 5 × 5 mm taking 2.62 s to acquire. The laser power on the cornea was <0.25 mW which is safe under the ANSI standard of ophthalmic instrument. After acquiring the raw interference signals, 3D data vis-OCT was generated by removing DC, converting the signals in k-space, digitally compensating the dispersion, and performing Fourier transform to prepare for later retinal boundary segmentation.

### Image Segmentation

We first detected the retinal pigmented epithelium (RPE) layer as the outer retina boundary. We blurred each B-scan by the Gaussian filter with a standard deviation of 7, then identified the RPE depth by detecting the maximum intensities along the A-line. Several factors can influence the accuracy of detection results, including the vessel shadows, high intensities in the retinal nerve fiber layer (RNFL) and system noise. To address these factors, we performed a correction by using a two-step outlier detection and replacement, as detailed below.

**Figure 1:**
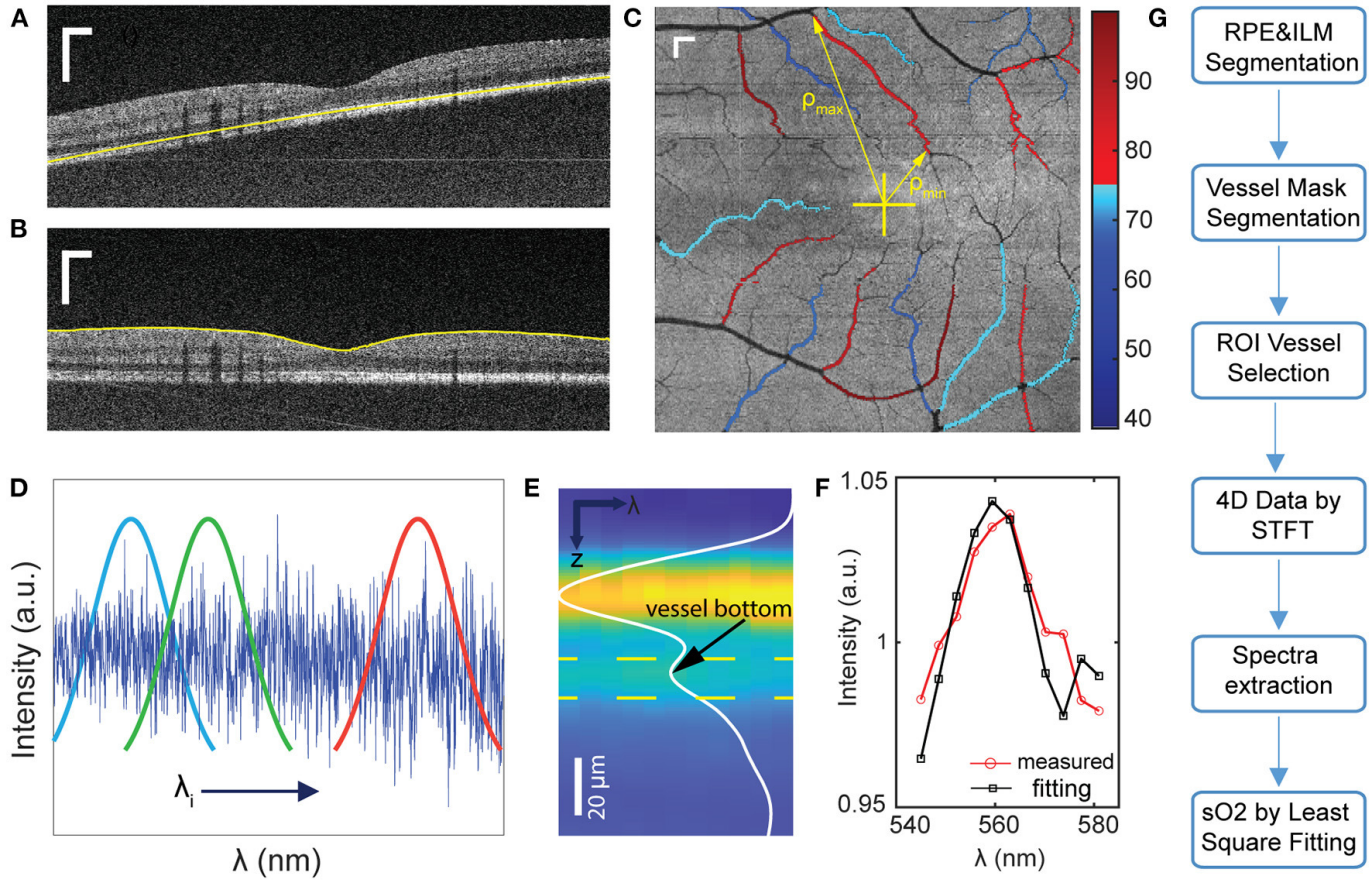
Illustration of the data processing workflow for sO_2_ calculation. **(A,B)** Examples of image segmentation for RPE detection, and (b) retina flattening and ILM layer detection. **(C)** Illustration of sO_2_ mapping of parafoveal vessels. ρ_min_ and ρ_max_ is the minimum and maximum distance from each vessel segment to fovea. **(D,E)** Short time Fourier transform to extract depth-resolved spectra. Yellow dashlines indicated the depth range for spectra extraction **(F)** sO_2_ regression by least square fitting. **(G)** Flow chart of data processing procedures. Scale bar: 250 μm.

We used a third-order polynomial to fit the detected RPE curve within each B-scan, then calculated the difference between the fitted and detected curves. The mean and standard deviation (SD) of the difference were obtained. When the difference is outside the range of mean ±1.8 × SD, this point on the detected RPE curve was considered as an outlier and deleted. Then we used the remaining points to generate the new polynomial fitting curve to replace the outliers and iterated this process until no outlier was found. We also calculated the coefficient of variation (COV) of each final fitted curve within each B-scan. After processing all B-scans, we obtained a COV curve over all B-scans, then we performed the outlier detection in the COV curve using the same standard to find the outliers. The values of COV of each fitting curve in adjacent images is expected to be similar. If the COV of two adjacent curves changed greatly in some frames, that usually mean the segmentation failed, and we discarded the outlier curve and used the previous curve to replace it. After this method, the retina was flattened based on accurate RPE labeling as shown in Figures 1A,B. In the next step, the inner limiting membrane (ILM) can be easily determined by the greatest gradient changes of intensities in the region above the RPE (Figure 1B).

We averaged the signals 20 pixels above RPE to generate the *en face* image. The vessel mask was obtained by binarizing the *en face* image with an adaptive threshold (23). Based on this vessel mask, we manually selected the vessel in the region of interest (ROI) as illustrated in Figure 1C. We considered the vessels branching from grandparents, to parents, and to children. Because of the strong blood absorption in VIS-OCT, the large grandparent vessels can be challenging to locate the vessel bottom and lack regional specification. Therefore, we focused on smaller parafoveal vessels and stopped at major branching points. When the two vessels were intersecting, we avoided selecting the overlaying portions. Tiny children vessels were also neglected as the adaptive threshold algorithm failed to recognize them.

### Oxygen Saturation Calculation

We generated the wavelength-dependent four-dimensional (4D) data I (*x, y, z*, λ) by the Short-time Fourier transform with 11 Gaussian windows sweeping the spectral interferogram (Figure 1D). We flatten all A-lines within each vessel ROI with respect to ILM and averaged all the A-lines of 4D data to generate the spectra in terms of depth I (*z*, λ) (Figure 1E). These spectra were then normalized by the averaged spectrum from non-vascular RNFL (22). To locate the vessel bottom, we averaged over λ and obtained an averaged A-line signal as the curve shown in Figure 1E. The location of the second reflective peak from ILM is identified as the vessel bottom. We then averaged the signals in the range of 5 pixels above and 10 pixels below the vessel bottom (area between the two yellow dash line in Figure 1E) in I (*z*, λ) to generate the single spectrum (Figure 1F).

Finally, we used a least square fitting on the extracted spectra to calculate the sO_2_ of ROI vessels. The model and algorithm were clarified in our previous work (22).


(1) $$\begin{array}{l} I\left(sO_{2}|\lambda ,z\right)=I_{0}\left(\lambda \right)\sqrt{R_{0}r\left(\lambda \right)}e^{-\left[sO_{2}\times \mu_{HbO_{2}}\left(\lambda \right)+\left(1-sO_{2}\right)\times \mu_{Hb}\left(\lambda \right)\right]z} \end{array}$$

Where *I*_0_(λ) is the spectrum of light sources; *R*_0_ is the reflectance of reference arm which is assumed to be constant; *r*(λ) (dimensionless) is the reflectance at the vessel wall, modeled by a power law *r*(λ) = *Aλ*^−α^, with A being a dimensionless constant and α modeling the decaying scattering spectrum from the vessel wall. Both *A* and α were included in the spectral fitting process. The detailed calculation for *r*(λ) can be found in reference (22). For the parameters μ_*HbO*_2__ and μ_*Hb*_, the optical attenuation coefficient μ is determined by the coefficients of absorption μ_*a*_ and scattering μ_*s*_, where. μ(λ) = μ_*a*_(λ)+*Wμ*_*s*_(λ). *W* is the scaling factor for the scattering coefficient which was 0.2 used here (8).

### Vessel Topographic Feature Analysis

Several topographic features of manually selected vessels were quantified, including length (*L*), area (*A*), diameter (*D*), the minimum distance (ρ_*min*_) and maximum distance (ρ_*max*_) from fovea to vessel segments. Based on the *en face* images, the length and area were defined as the converted length of vessel centerline and area of whole vessel ROI segment. The diameter *D* was obtained by top and bottom of the vessels from averaged A-line signals. The accurate location of fovea was targeted manually by going through the 3D data combined with the *en face* image. With the known fovea location and selected vessel masks, we calculated ρ_*min*_ *and* ρ_*max*_ *for* each vessel segment.

### Statistical Analysis

Statistical analysis was performed by MATLAB (The MathWorks, Inc.). Pearson's linear correlation coefficients were calculated between each pair of parameters, and unpaired parametric t-statistics were executed for the *p*-values. When the *p* < 0.05, the statistics was defined as the significant.

## Results

Fourteen healthy subjects in total were recruited in this study. Four subjects were excluded due to the failure of fixation, or low image quality. Fixation failures were due to significant eye movements during imaging where fovea was not present in the images, or the vessel locations were not recognizable. Lower image qualities caused the unsuccessful segmentation, leading to the failure of vessel selection and sO_2_ calculation. Additional four eyes were excluded by the same criterion within the left ten subjects. The demographic information and the ophthalmic measurements are summarized in Tables 1, 2. Sixteen eyes in total were used for the analysis. Nine eyes have mild Grade 1 cataract.

**Table 1:** Demographic information of subjects.

**Subject numbers**	**10**
Gender (female/male)	5/5
Eyes (OD/OS)	8/8
Ages (years)	61 ± 13.3
Race (White/AA/NA)	3/4/3
Ethnicity (Not Latino/Latino/NA)	7/2/1

*AA, African American; NA, Not available*.

**Table 2:** Characteristics of ocular measurements from all eyes.

**Characteristics**	**Mean (std)**
Sphere (Diopter)	−0.50 (1.53)
Cylinder (Diopter)	0.77 (0.97)
IOP (mmHg)	15.4 (2.3)
CDR	0.33 (0.09)
GCC (μm)	78.0 (8.9)
cpRNFL (μm)	90.7 (10.0)
Superior cpRNFL (μm)	115.9 (14.0)
Nasal cpRNFL (μm)	70.3 (14.9)
Inferior cpRNFL (μm)	116.6 (17.4)
Temporal cpRNFL (μm)	61.6 (10.3)
AsO_2_ (vol %)	92.1 (7.1)
VsO_2_ (vol %)	48.4 (5.0)
A-V sO_2_ (vol %)	43.8 (9.5)

**Figure 2:**
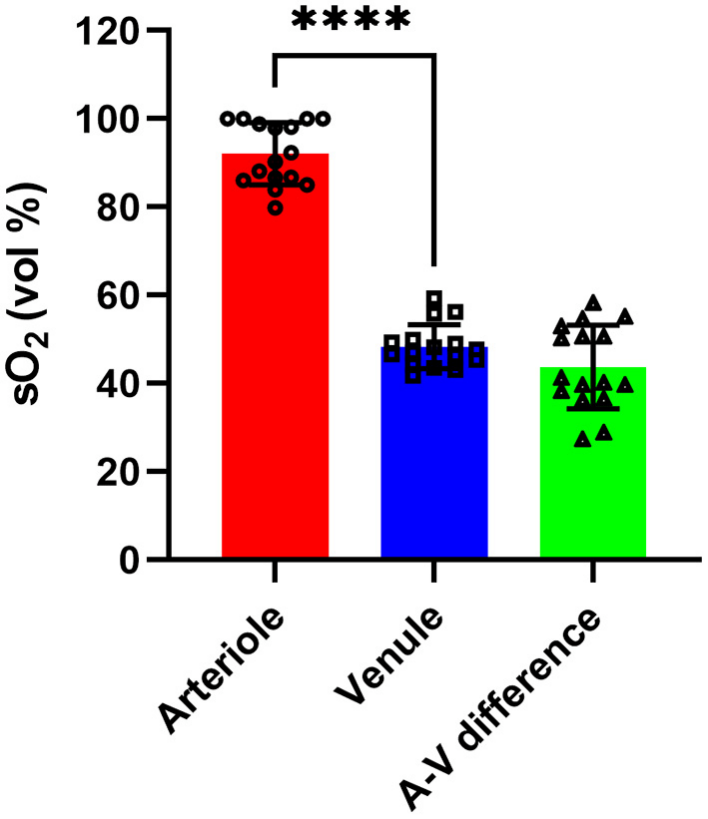
The sO_2_ of arterioles, venules, and A-V sO_2_ difference from all eyes. ****p < 0.0001.

The sO_2_ value for all arterioles and venules were first averaged within each eye, as shown in Figure 2. The mean arterial (AsO_2_) and venous sO_2_ (VsO_2_) were 92.1 ± 7.1 (vol %) and 48.4 ± 5.0 (vol %) respectively. The mean arteriovenous sO_2_ difference (A-V sO_2_) was 43.8 ± 9.5 (vol %). The mean and standard deviation of vessel sO_2_ for both arterioles and venules from all eyes are shown in Supplementary Figure 3.

**Figure 3:**
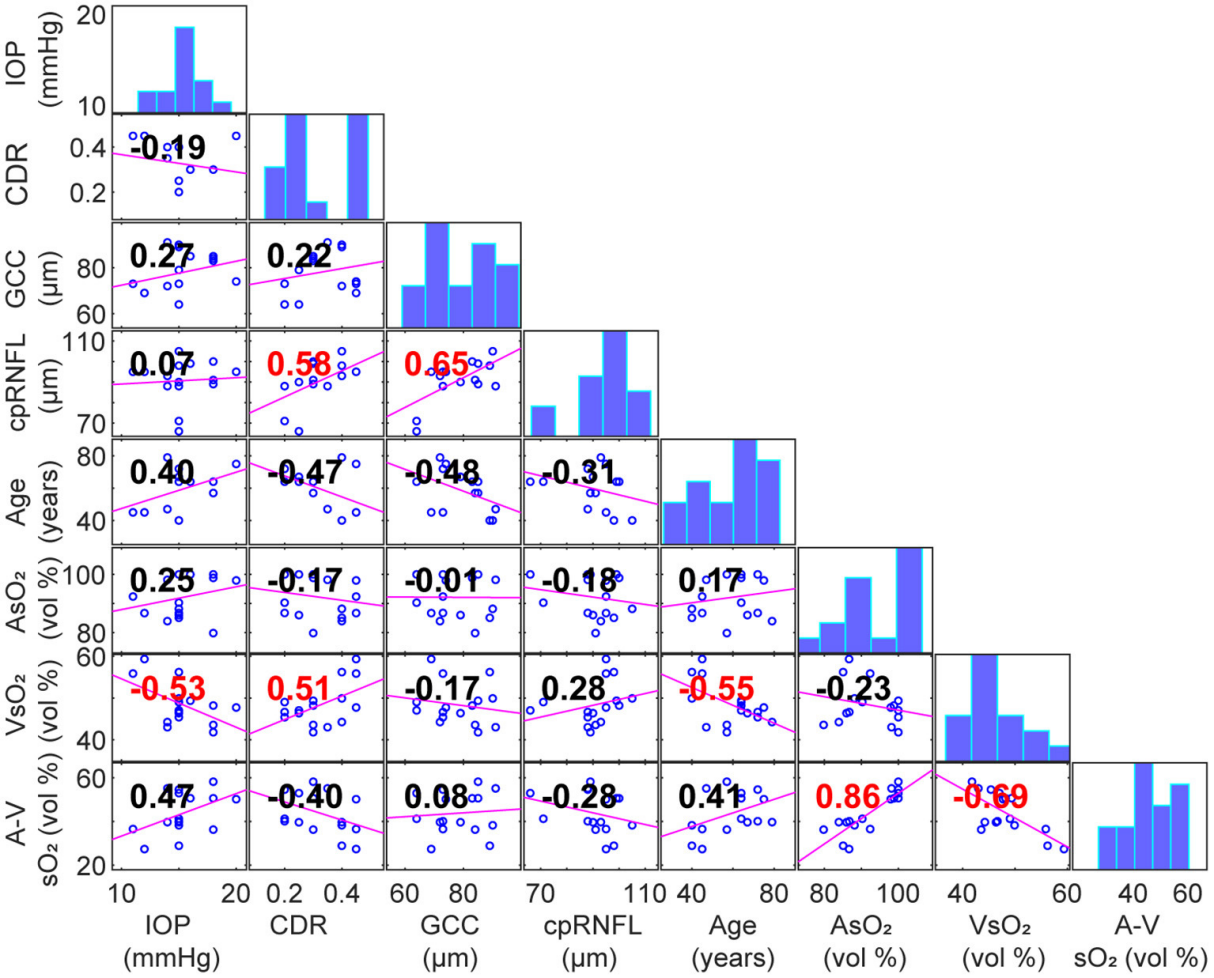
Scatterplot with linear fit for a pair of correlated variables (Panels below the diagonal. Correlation coefficients area labeled in panels. Red: *p* < 0.05). Diagonal panels are histograms of each variable.

Table 3 summarizes the correlation coefficients and *p* values between parafoveal AsO_2_, VsO_2_, A-V sO_2_ and ophthalmic measurements including refractive errors, IOP, thickness of macular ganglion cell complex (GCC) and circumpapillary RNFL (cpRNFL). No significant correlation was found except between VsO_2_ and IOP, cup/disk ratio (CDR) and age. Figure 3 shows the correlation matrix map between sO_2_ per eye and other ophthalmic parameters.

**Table 3:** Correlation between clinical parameters and vessel sO_2_ based on eyes.

**Clinical Parameter**	**AsO**_**2**_		**VsO**_**2**_		**A-V sO**_**2**_	
	***R***	***p***	***r***	***p***	***r***	***p***
Sphere	−0.185	0.492	−0.368	0.160	0.055	0.838
Cylinder	0.195	0.524	0.184	0.546	0.045	0.884
IOP	0.253	0.344	−0.530	**0.035**	0.466	0.069
CDR	−0.173	0.521	0.514	**0.042**	−0.398	0.127
GCC	−0.007	0.980	−0.168	0.534	0.083	0.760
cpRNFL	−0.180	0.504	0.283	0.288	−0.282	0.291
Age	0.174	0.520	−0.546	**0.029**	0.414	0.110

*Pearson's correlation was performed. Bold: p < 0.05*.

We further analyzed the correlation between sO_2_ and vessel topographic parameters, which were in the Supplementary Figures 1, 2. No significant correlation was found between sO_2_ and vessel length (*L*), area (*A*), diameter (*D*), and the distance of ρ_min_ and ρ_max_.

For the venules, *A* and *L* is significantly correlated as expected by geometry. The diameter positively correlated with the length and area of venules but with no significance. The minimum distance (ρ_min_) has a negative correlation with length, area, and diameter, but only has a significant dependency on the latter two. The maximum distance (ρ_max_) has significant dependencies on all the other dimensions except diameter. For the arterioles, sO_2_ has no significant correlation with topographic parameters. All the topographic parameters have significant correlation with each other, except for the ρ_min_ with length, area, and diameter.

Table 4 summarizes the topographic parameters and sO_2_ values from all of arterious and venous segments. The AsO_2_ and VsO_2_ per segments are 91.0 ± 8.0 vol % (*n* = 57) and 50.9 ± 8.8 vol % (*n* = 43), and the diameter of venules is significantly larger than the arterioles' (*P* < 0.001). For the other parameters, no significant difference was found. It is noted that diameters of both arterioles and venules are smaller than 40 μm.

**Table 4:** Vessel segment topographic parameters and sO_2_ (*n* = 57 for arterioles, *n* = 43 for venules).

	**Arterioles**	**Venules**	***p***
L (mm)	1.19 ± 0.48	1.23 ± 0.42	0.709
A (10^−3^ mm^2^)	32.5 ± 17.3	37.1 ± 16.6	0.184
*D* (μm)	24.8 ± 3.9	28.4 ± 3.2	**<0.001**
ρ_min_ (mm)	1.11 ± 0.39	1.16 ± 0.53	0.604
ρ_max_ (mm)	2.06 ± 0.51	2.26 ± 0.52	0.061
sO_2_ (vol %)	91.0 ± 8.0	50.9 ± 8.8	**<0.001**

*Two sample t-tests. Bold: p < 0.05*.

## Discussion

In this paper, we report the first baseline study on parafoveal vessel oxygen saturation (sO_2_) in healthy eyes. We quantified the topographic features of those vessels and found no significant correlation with sO_2_ values. There is no significant correlation between sO_2_ and cpRNFL, GCC thickness or refractive error. IOP moderately correlated with VsO_2_; however, A-V sO_2_ had no significant correlation with IOP. The significant association between VsO_2_ and age and CDR was also found.

We found the negative correlation between VsO_2_ and age in healthy subjects was significant, which is consistent with previous reports (24–26). We also found correlation between VsO_2_ and IOP. There has been sparse literature investigating the association between VsO_2_ and IOP. Liu et al. reported no association between IOP and sO_2_ for the healthy children under 18 (27). However, there is a significant age difference in our study and thus it is difficult to compare. It is plausible that IOP was positively correlated to the age of people under 60 (28), leading to the negative correlation to the VsO_2_. The positive association between VsO_2_ and CDR was marginally significant, which can also be found in Vandewalle et al. (29). Despite above significant correlation, we found no significant correlation between A-V sO_2_ with age, IOP or CDR.

Compared to fundus-based or multi wavelength SLO oximetry (7, 30), VIS-OCT provides better depth resolution enabling precise segmentation from the bottom of the vessels, and avoids noise from other layers. The parafoveal vessels in this study have a modest diameter ranging from 20 to 30 μm where visible light may penetrate fully. Without the depth segmentation, the penetrated light would diffuse through other layers and create confounding factors in the fundus camera. This would also compromise the resolution and contrast and make macular oximetry challenging for 2D image-based methods. We also note that the relative smaller caliber of the parafoveal vessels may be better suited for VIS-OCT than larger peripapillary vessels around ONH, since the bottom of the vessels were easier to image than large vessels to create a strong reflectance signal.

While this is the first study of the parafoveal vessel sO_2_ in normal human subjects using VIS-OCT oximetry, there are limitations worth noting. First, we had limited subject number in this pilot study. Therefore, we refrained from advanced multivariable statistical analysis and rather performed simple two-sample *t*-tests and Pearson's linear correlation. Marginal significances were found in some parameters stated above. A larger cohort would be needed in the future study to validate those significance by a multiple-variable analysis to incorporate variable interactions and other confounding factors. Second, there are the technical limitations that we would like to improve in the future. The spectral bandwidth in the current device is limited in exchange for large imaging depth for better alignment. With our new linear-K spectrometer design and extended imaging depth (31), the spectral bandwidth can be increased to improve the robustness and accuracy of sO_2_ calculation.

In summary, we investigated parafoveal microvascular sO_2_ using VIS-OCT on healthy subjects in this initial baseline study. The statistical analysis reveals that no significant association between sO_2_ and vessel topographic features. Also, there are no significant correlations for the eye sO_2_ and clinical parameters except the relation of VsO_2_ and IOP, CDR and age. This study will provide a normal (an initial) database for later sO_2_ investigations in ocular diseases.

## Data Availability Statement

The raw data supporting the conclusions of this article will be made available by the authors, without undue reservation.

## Ethics Statement

The studies involving human participants were reviewed and approved by Boston Medical Center. The patients/participants provided their written informed consent to participate in this study.

## Author Contributions

JY supervised this project. JW and JY analyzed the results. JW, WS, MF, MD, and JY contributed the manuscript. WS, NS, MF, and MD assisted in subject screening and recruitment. All authors contributed to the article and approved the submitted version.

## Funding

NEI/NINDS R01NS108464, R01 EY032163 and R21EY029412.

## Conflict of Interest

The authors declare that the research was conducted in the absence of any commercial or financial relationships that could be construed as a potential conflict of interest.

## Publisher's Note

All claims expressed in this article are solely those of the authors and do not necessarily represent those of their affiliated organizations, or those of the publisher, the editors and the reviewers. Any product that may be evaluated in this article, or claim that may be made by its manufacturer, is not guaranteed or endorsed by the publisher.
