# Supplementary material for: A Baseline Study of Oxygen Saturation in Parafoveal Vessels Using Visible Light Optical Coherence Tomography
Source: Front Med (Lausanne). 2022 May 12;9:886576. doi: 10.3389/fmed.2022.886576 (PMC9133487; doi:10.3389/fmed.2022.886576)
Supplement: Supplementary file 1 [file Data_Sheet_1.docx]

Supplementary Material

## Supplementary Figures
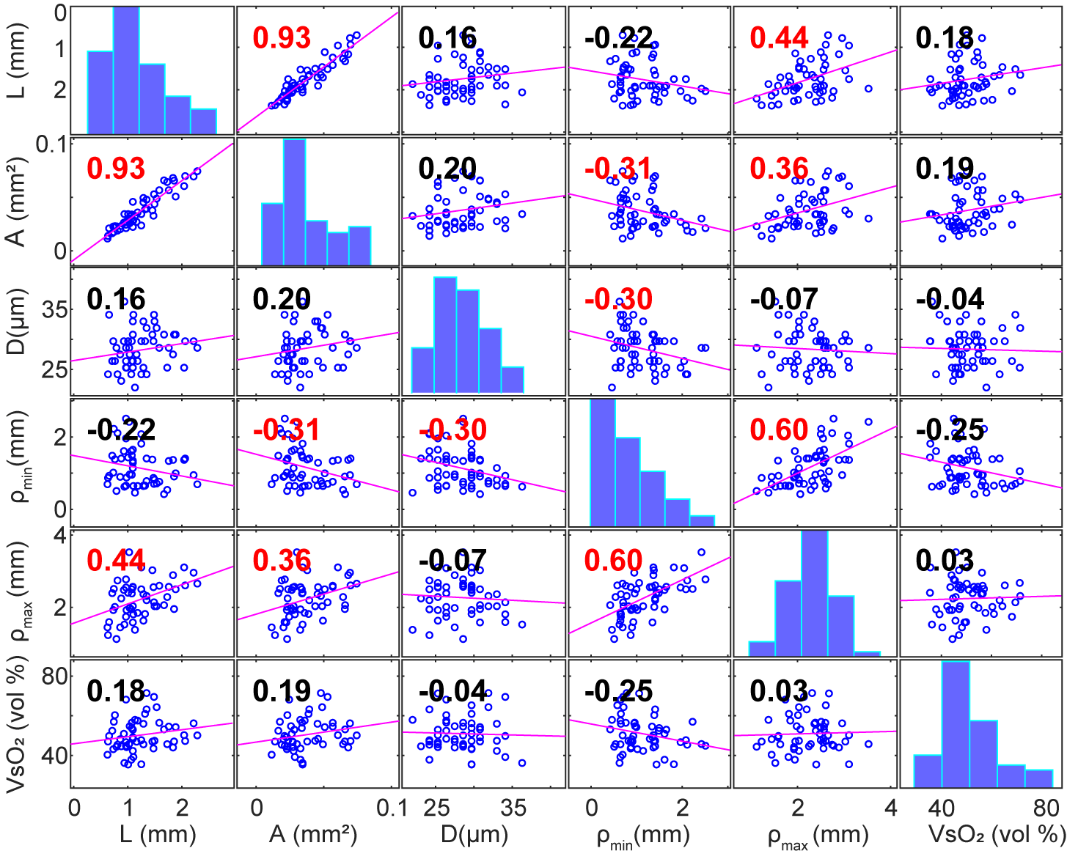


## Figure S1. Correlation map of vessel topography parameters and venous sO2 (Correlation coefficients are red when p< 0.05).


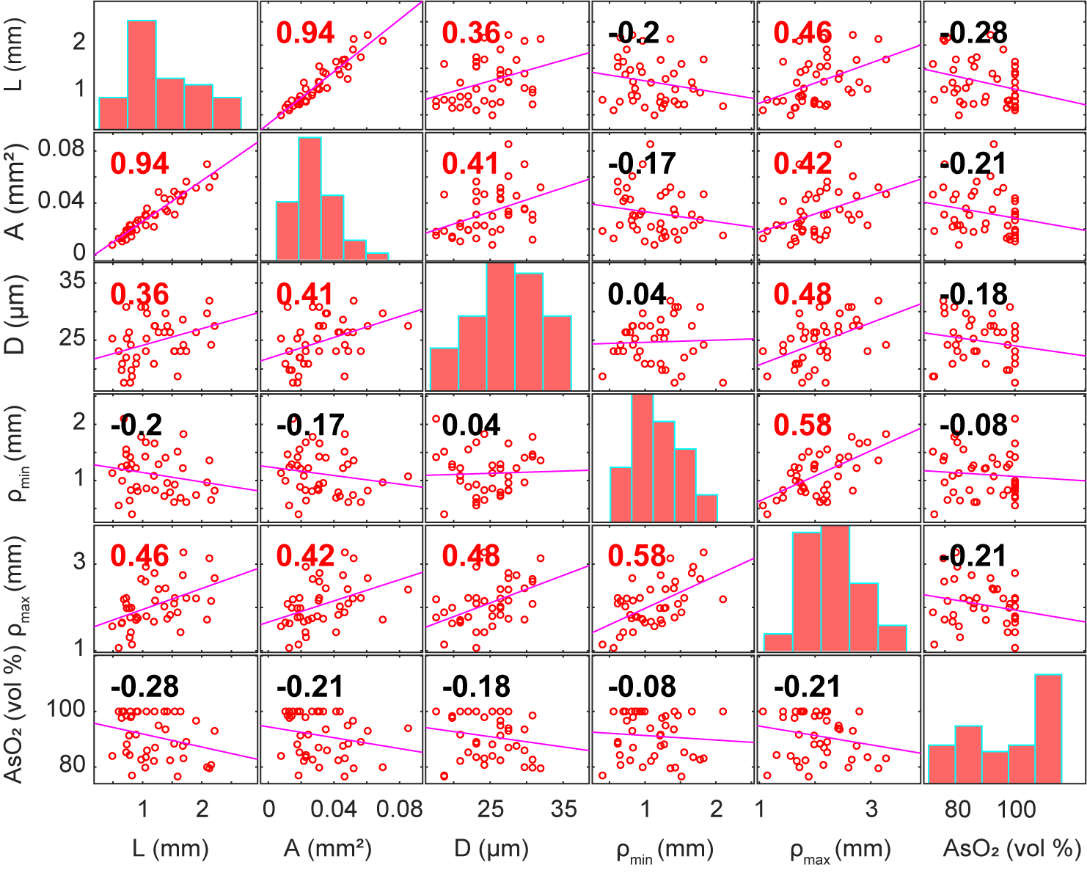


Figure S2. Correlation map of vessel topographic parameters and arterious sO2 of arterioles (Correlation coefficients are red when p <0.05).


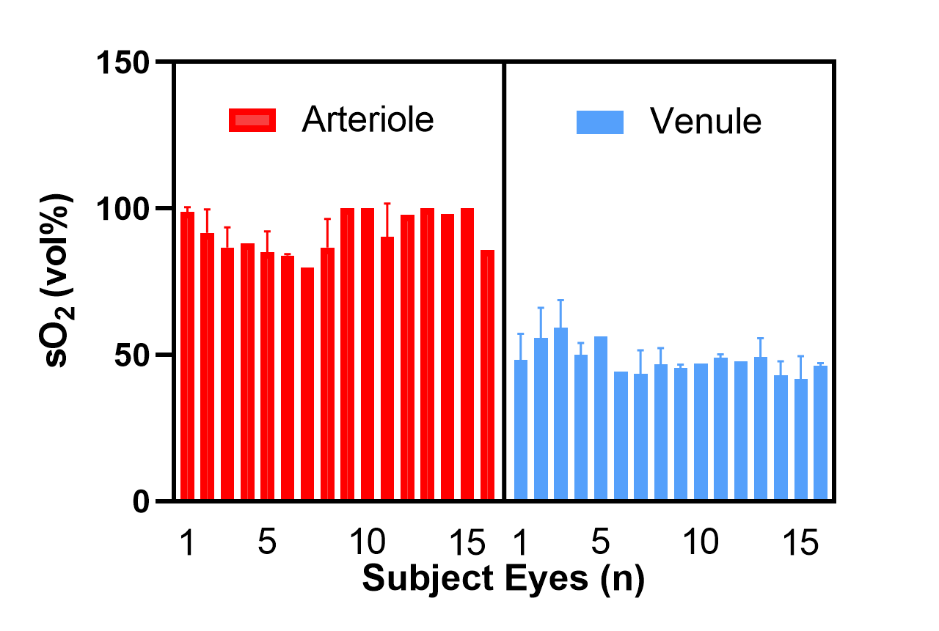


Figure S3 The mean and standard deviation of vessel sO2 in all subject eyes.
